# Factors associated with Community Health Agents’ knowledge about tuberculosis

**DOI:** 10.1590/0034-7167-2022-0520

**Published:** 2024-05-13

**Authors:** Clery Mariano da Silva Alves, Tauana de Souza Amaral, Fabiana Ribeiro Rezende, Hélio Galdino, Rafael Alves Guimarães, Dayane de Melo Costa, Anaclara Ferreira Veiga Tipple

**Affiliations:** I Universidade Federal de Goiás. Goiânia, Goiás, Brazil

**Keywords:** Community Health Workers, Tuberculosis, Knowledge, Occupational Health, Occupational Exposure, Agentes Comunitarios de Salud, Antituberculosos, Conocimiento, Salud Laboral, Exposición Profesional, Agentes Comunitários de Saúdes, Tuberculose, Conhecimento, Saúde do Trabalhador, Exposição Ocupacional

## Abstract

**Objective:**

To analyze the factors associated with the knowledge of Community Health Agents (ACS) about tuberculosis.

**Methods:**

A cross-sectional study was conducted with 110 ACS. A questionnaire was used to assess knowledge about pulmonary tuberculosis (component 1) and the work functions of ACS in the National Tuberculosis Control Program (component 2). The level of knowledge, according to the scores converted into a scale of 0 to 100, was classified as: 0-50% (low), 51-75% (medium), and over 75% (high). Multiple regression was used in the analysis of associated factors.

**Results:**

The global score (average of the scores of components 1 and 2) median knowledge was 68.6%. Overall knowledge about tuberculosis was positively associated with the length of professional experience, having received training on tuberculosis, and access to the tuberculosis guide/handbook.

**Conclusions:**

Investments in training and capacity-building strategies for ACS will contribute to increasing these professionals’ knowledge, resulting in greater success in tuberculosis control.

## RESUMO

**Objetivo:**

Analisar os fatores associados ao conhecimento dos Agentes Comunitários de Saúde (ACS) sobre tuberculose.

**Métodos:**

Estudo transversal realizado com 110 ACS. Utilizou-se um questionário para avaliar o conhecimento sobre tuberculose pulmonar (componente 1) e as funções laborais dos ACS no Programa Nacional de Controle de Tuberculose (componente 2). O nível de conhecimento, de acordo com os escores transformados em uma escala de 0 a 100, foi classificado como: 0-50% (baixo), 51-75% (mediano) e mais de 75% (alto). A regressão múltipla foi empregada na análise dos fatores associados.

**Resultados:**

A pontuação global (média dos escores dos componentes 1 e 2) mediana do conhecimento foi de 68,6%. O conhecimento global sobre tuberculose mostrou-se positivamente associado ao tempo de atuação profissional, à recepção de capacitação sobre tuberculose e ao acesso ao guia/cartilha de tuberculose.

**Conclusões:**

Investimentos na formação e nas estratégias de capacitação dos ACS contribuirão para o aumento do conhecimento desses profissionais, resultando em maior sucesso no controle da tuberculose.

## RESUMEN

**Objetivo:**

Analizar los factores asociados al conocimiento de los Agentes Comunitarios de Salud (ACS) sobre la tuberculosis.

**Métodos:**

Se realizó un estudio transversal con 110 ACS. Se utilizó un cuestionario para evaluar el conocimiento sobre la tuberculosis pulmonar (componente 1) y las funciones laborales de los ACS en el Programa Nacional de Control de la Tuberculosis (componente 2). El nivel de conocimiento, según las puntuaciones convertidas en una escala de 0 a 100, se clasificó como: 0-50% (bajo), 51-75% (medio) y más del 75% (alto). La regresión múltiple se utilizó en el análisis de factores asociados.

**Resultados:**

La puntuación global (media de las puntuaciones de los componentes 1 y 2) del conocimiento mediano fue del 68,6%. El conocimiento general sobre la tuberculosis estuvo positivamente asociado con la duración de la experiencia profesional, haber recibido capacitación sobre tuberculosis y el acceso a la guía/manual de tuberculosis.

**Conclusiones:**

Las inversiones en formación y estrategias de capacitación para los ACS contribuirán a aumentar el conocimiento de estos profesionales, lo que resultará en un mayor éxito en el control de la tuberculosis.

## INTRODUCTION

Tuberculosis is a communicable disease of global epidemiological importance, caused by *Mycobacterium tuberculosis* ^(1)^. In 2021, it was estimated that approximately 10 million people were infected by this bacillus ^(2)^. A meta-analysis indicates that healthcare workers have a higher risk of developing tuberculosis compared to the general population ^(3)^.

In response, the World Health Organization has developed goals to reduce the incidence of this disease in the population ^(2)^. In Brazil, tuberculosis control is conducted through the National Tuberculosis Control Program (PNCT), which is based on care models included in the Family Health Strategy (FHS) ^(4)^. Jesus et al.^(5)^ report the effectiveness of this program in the country, finding that the FHS is associated with lower incidence and mortality rates from tuberculosis^(5)^. The Community Health Agent (CHA) plays a significant role in this program, acting as a mediator between health services and users, contributing to the early diagnosis of tuberculosis^(4,6)^. Their functions include actively searching for cases, identifying individuals with respiratory symptoms, referring suspected cases to health services, and monitoring Directly Observed Treatment (DOT) during home visits ^(4,6)^.

However, due to their occupational activities, CHA are exposed to various risks, including biological risks such as exposure to saliva, a crucial factor in the transmission of *Mycobacterium tuberculosis* ^(7)^. Studies indicate a limited awareness among CHA about the risks they face, resulting in occupational vulnerability ^(8-10)^. Although satisfactory knowledge about tuberculosis has been found among CHA ^(11-13)^, there are still gaps in areas such as identifying patients with pulmonary tuberculosis ^(11,13)^, understanding the target audience for DOT, the proper technique for treatment supervision ^(11)^, and prevention ^(13)^. These areas should be included in the CHA training process, as their absence can compromise treatment and influence the adoption of occupational protection measures. Notably, CHA do not necessarily need a health background to perform their work, which affects the competencies required to provide various guidelines to the community they serve, including those related to tuberculosis.

Therefore, given that CHA perform essential activities for the quality of primary care^(14-17)^, characterizing the profile of CHA active in tuberculosis prevention and control, as well as elucidating factors related to their knowledge about tuberculosis and its protective measures, can inform strategies and/or public policy development for training these healthcare workers. This approach offers opportunities for successful measures in disease control and protection against the occupational biological risks faced by CHA.

## OBJECTIVE

To analyze the factors associated with the knowledge of Community Health Agents (CHA) about tuberculosis.

## METHODS

### Ethical Aspects

The research project was approved by the Research Ethics Committee of the Federal University of Goiás. Participants agreed to participate in the study by signing an Informed Consent Form, adhering to all the precepts of Resolution 466/2012^(18)^ of the National Health Council.

### Design, Period, and Location of the Study

This cross-sectional, analytical study was guided by the STrengthening the Reporting of OBservational studies in Epidemiology - STROBE tool and conducted in the city of Goiânia, Goiás, in the Central-West region of Brazil. In alignment with the decentralizing principle of healthcare, Goiânia is divided into seven regions, known as Health Districts (DS). The research was carried out in the West DS, which has 16 Family Health Centers (CSF) and ranks second in the number of CHA in the municipality. This DS was intentionally selected due to its size as the second-largest in the municipality and the higher availability of the CSF to participate in the study, thus allowing for a larger number of participants.

### Population and Inclusion and Exclusion Criteria

The target population of the study consisted of all (n=172) CHA from the Family Health teams of the 16 CSF in the West DS of Goiânia, Goiás, who were active during the data collection period and were aged 18 years or older.

### Study Protocol

Data collection was conducted using a self-administered questionnaire, developed according to the “Manual for the Community Health Agent - Tuberculosis”^(3)^ and the National Plan for the End of Tuberculosis as a Public Health Problem ^(2)^. The questionnaire, comprised of multiple-choice questions, was organized into four sections: demographic data, occupational data, knowledge about tuberculosis, and knowledge about the role of CHA in tuberculosis control.

This questionnaire was reviewed by three experts, each holding a doctoral degree and possessing knowledge in primary care and tuberculosis. They proposed several changes. The research team considered the suggestions and accepted all those recommended by at least two of the evaluators. Subsequently, a pilot test was carried out with 12 CHA working in two CSF of another municipality in the Goiânia metropolitan region, not included in the study. Adjustments were made to enhance the clarity of the questions. Following these stages, the questionnaire was deemed appropriate for achieving the study’s objectives.

Data collection occurred between July and December 2019, conducted by two nurses and three research assistants who were previously qualified. Initially, there was a meeting with the researchers to introduce the study, detailing its objectives, methodology, and questionnaire, and to discuss data collection strategies. Each research assistant observed the administration of the questionnaire by a researcher at least once.

Before the questionnaire was administered, telephone contact was made with the manager of each CSF to schedule the days and times when the CHA would be at the unit for joint activities, referred to as the “counter-shift.” On the appointed days, in a designated room, the CHA were briefed about the study’s objectives. Those who consented were given the Informed Consent Form, which they read and then signed in duplicate. The questionnaires were then distributed, with an estimated completion time of about 20 minutes.

As mentioned, an instrument was developed to measure the knowledge level of Community Health Agents (CHAs) regarding tuberculosis. It contained a total of 21 affirmative statements about the disease and 10 items about the job functions of CHAs in the National Tuberculosis Control Program (NTCP). For each item, the participant was required to mark ‘V’ (true) or ‘F’ (false). The 21 items about tuberculosis knowledge formed Component 1, and the 10 items about the job functions of CHAs in the NTCP formed Component 2.

The calculation of each participant’s knowledge score was conducted in three stages. In the first stage, a score of “1” was assigned for each correct answer and “0” for incorrect ones. In the second stage, the raw scores for each component were computed, corresponding to the sum of the total correct responses for each participant. The raw scores could range from zero to 21 for component 1 (knowledge about tuberculosis), from zero to 10 for component 2 (knowledge about the occupational functions of CHA in the PNCT), and from 0 to 31 for a global knowledge component on tuberculosis, obtained by summing the raw scores of both components. In the third stage, the scores were transformed into a scale of 0 to 100, by dividing the raw score by the maximum possible total score for the component and multiplying by 100. For example, if an CHA obtained a raw score of 11 points in component 1, their knowledge would be 52.4%, as (11/21)*100 = 52.4%. This generated a knowledge score that varied from 0 to 100%, with 0 being the lowest and 100 the highest knowledge level of the CHA in each component.

Knowledge in each analyzed aspect (component 1, component 2, and global knowledge) was classified, based on the scores generated in the last stage, into: low knowledge (0 to 50% score), medium knowledge (50 to 75% score), and high knowledge (more than 75% score).

Three dependent variables were considered: (i) the knowledge score of CHA on pulmonary tuberculosis (component 1), considered as a continuous variable on a scale of 0 to 100%; (ii) knowledge score about the occupational functions of CHA in the PNCT (component 2), also considered as a continuous variable from 0 to 100%; and (iii) global knowledge score, which included the arithmetic mean of the scores of components 1 and 2, considered as a continuous variable on a scale of 0 to 100%.

The independent variables were: gender, age, education, length of service, work hours, participation in tuberculosis training, access to a tuberculosis guide/manual, supervision of people with tuberculosis, the number of people with tuberculosis supervised, supervision of DOT, and supervision of the quantity of patients under DOT.

### Analysis of Results and Statistics

The data analysis was conducted using Stata software, version 15.0. Initially, the Kolmogorov-Smirnov (K-S) test with Lilliefors correction was applied to verify the normality of quantitative variables. Subsequently, a descriptive analysis of all study variables, including those related to knowledge, was conducted. Qualitative variables were presented in terms of absolute frequency (n) and relative frequency (%), and quantitative variables in terms of median, 25th percentile (P25), 75th percentile (P75), as well as minimum and maximum values, due to the absence of normality observed in the K-S test. For the knowledge questions assessed, a 95% confidence interval (CI95%) was also estimated.

The dependent variables included in the regression analysis were continuous, on scales of 0 to 100%, as previously mentioned. Therefore, simple and multiple linear regression analyses were employed to examine factors associated with knowledge scores, considering the continuous nature of these variables. Initially, a bivariate analysis was conducted using simple linear regression to assess the magnitude of the association between each independent variable and the investigated dependent variables. The results of this analysis were presented as the regression coefficient (β) and respective CI 95%. In a second stage, variables with a p-value less than 0.20 and potential confounders (such as age, gender, and length of professional service) were included in multiple linear regression models, using a single-entry method. The results of these statistical models were displayed as the regression coefficient (β), respective CI 95%, and standardized regression coefficient (βp). The statistical significance of the analyses was evaluated by the t-test.

The multiple linear regression models were assessed and validated regarding their assumptions, including the absence of multicollinearity, verified by the Variance Inflation Factor (VIF), as well as linearity, homoscedasticity, and normality of residuals. Variables with a p-value less than 0.05 were considered statistically significant.

## RESULTS

Among the 172 Community Health Agents (CHA) working in the evaluated district, 110 responded to the questionnaire, representing a response rate of 64.0%. The median age of the participants was 40 years (P25=34.0; P75=46.3; minimum=26; maximum=61), with 47.3% of the sample being up to 39 years old. The majority of participants were female (91.8%), self-identified as mixed race/brown (66.4%), were married or in a common-law partnership (71.8%), and had completed high school (58.2%). The median tenure as an CHA was 6.1 years (P25=3.8; P75=10; minimum=1; maximum=20 years), and 29.1% of the sample had 10 or more years of service. Holding more than one job was reported by 9.1% of the CHA.

Of the respondents, 65 (59.1%) declared having experience in accompanying individuals with tuberculosis during their professional tenure, and 34 (52.3%) of them reported having supervised Directly Observed Treatment (DOT), with the majority (33 or 97.9%) conducting one to five supervisions, typically once or twice a week (29 or 88.2%).

Regarding protective measures used during the supervision of DOT, 64.7% (22/34) mentioned hand hygiene. The use of PFF2 (N95) respirators and gloves was reported by 8.8% (3/34) of the participants, while none mentioned using protective eyewear.

In terms of access to educational materials and training, 54.5% (60/110) of the CHA reported having access to a tuberculosis guide/manual, and 46.4% (51/110) participated in training on the disease. The most addressed topics in these trainings were signs and symptoms of tuberculosis (100% or 51/51), active search for respiratory symptomatics (88.2% or 45/51), and the roles of CHA in tuberculosis control (88.2% or 45/51). The least covered topics were anti-tuberculosis drugs and their side effects (54.9% or 28/51), supervision of DOT (52.9% or 27/51), and tuberculosis control in Primary Care (45.1% or 23/51). Protective measures against tuberculosis were indicated by 70.6% (36/51) of the participants.

**Figure 1:**
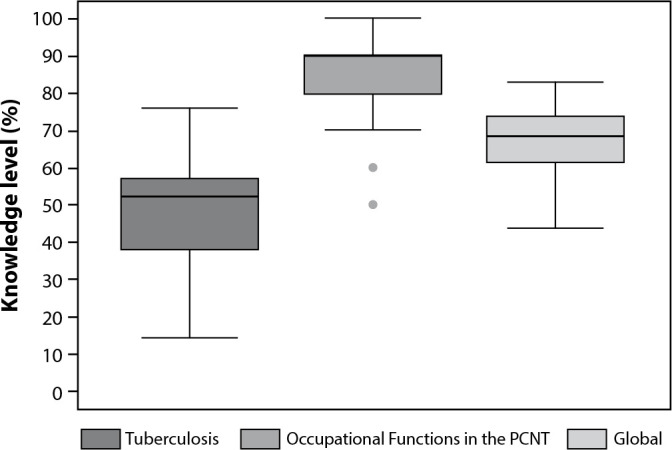
Knowledge about Tuberculosis, Occupational Functions in the National Tuberculosis Control Program (PNCT), and Global Knowledge by Community Health Agents in a Health District of the Municipality of Goiânia, Goiás, Brazil, 2019

The median knowledge score related to tuberculosis was 52.4% (P25=34.0; P75=57.1; minimum=14.3; maximum=76.2), and the knowledge score about occupational functions in the PNCT was 90.0% (P25=80.0; P75=90.0; minimum=50.0; maximum=100.0). The global knowledge score, which corresponds to the average of the two components, had a median of 68.6% (P25=61.4; P75=73.6; minimum=44.1; maximum=83.1) (Figure 1).

The results also showed that, regarding component 1 (knowledge about tuberculosis), 49.1% of participants had low knowledge, 49.1% had medium knowledge, and 1.8% had high knowledge. As for component 2 (knowledge about the occupational functions of CHA in the PNCT), 0.9% had low knowledge, 12.7% had medium knowledge, and 86.4% had high knowledge. Concerning global knowledge, 4.5% of participants had low knowledge, 72.7% had medium knowledge, and 22.7% had high knowledge.

The analysis showed that the longer the tenure as a Community Health Agent (CHA), the greater the global knowledge (β=2.36; p=0.028) and knowledge about tuberculosis (β=4.38; p=0.005). Also, having undergone training on tuberculosis was associated with greater global knowledge (β=7.24; p<0.001), knowledge about tuberculosis (β=10.85; p<0.001), and knowledge about the occupational functions of CHA in the PNCT (β=3.72; p=0.041). Similarly, access to a tuberculosis guide/manual was associated with greater global knowledge (β=4.86; p=0.005) and knowledge about tuberculosis (β=6.68; p=0.008). A positive association was also found between experience in supervising Directly Observed Treatment (DOT) and knowledge about tuberculosis (β=5.93; p=0.030) in the bivariate analysis.

The final model analysis of factors associated with the CHA’s knowledge showed positive associations between length of professional service and global knowledge (β=2.43; p=0.027) and knowledge about tuberculosis (β=4.63; p=0.003) (Table 1). As the tenure as an CHA increased, global knowledge increased by 2.43% and knowledge about tuberculosis increased by 4.63%.

**Table 1:** Multiple Regression Analysis of Factors Associated with Knowledge about Tuberculosis (TB) and Occupational Functions in the National Tuberculosis Control Program, and Global Knowledge by Community Health Agents in a Health District of the Municipality of Goiânia, Goiás, Brazil, 2019

Variables	β	IC 95%	β_p_	*t*	*p* value
Global					
Gender:					
Male (Reference)					
Female	1.26	-4.70; 7.20	0.04	0.42	0.676
Age (years)	-0.14	-0.35; 0.08	-0.12	-1.27	0.206
Years of Service (years)	2.43	0.29; 4.57	0.22	2.25	0.027
Training on Tuberculosis (TB):					
No (Reference)					
Yes	6.16	2.95; 9.37	0.34	3.81	**<0.001**
Access to the Guide:					
No (Reference)					
Yes	3.59	0.44; 6.74	0.20	2.26	0.026
Supervised DOT†:					
No (Reference)					
Yes	-0.87	-4.78; 3.03	-0.04	-0.45	0.657
Component 1					
Gender:					
Male (Reference)					
Female	-0.51	-9.89; 8.86	-0.01	-0.11	0.914
Age (years)	-0.18	-0.49; 0.13	-0.10	-1.13	0.260
Years of Service (years)	4.63	1.57; 7.68	0.28	3.00	0.003
Component 1					
Training on TB:					
No (Reference)					
Yes	9.62	4.87; 14.36	0.36	4.02	**<0.001**
Access to the Guide/Manual:					
No (Reference)					
Yes	5.64	0.38; 10.91	0.21	2.12	0.036
Supervised DOT†:					
No (Reference)					
Yes	1.55	-3.31; 6.40	0.05	0.63	0.530
Component 2					
Gender:					
Male (Reference)					
Female	3.24	-3.27; 9.75	0.09	0.99	0.326
Age (years)	-0.06	-0.29; 0.15	-0.06	-0.59	0.555
Years of Service (years)	-0.53	-2.72; 1.67	-0.04	-0.48	0.634
Training on TB:					
No (Reference)					
Yes	3.48	0.18; 6.79	0.18	2.09	0.039
Access to the Guide:					
No (Reference)					
Yes	2.23	-1.36; 5.82	0.12	1.81	0.220

*Component 1: Score of the Community Health Agents’ (CHAs) knowledge about pulmonary tuberculosis; Component 2: Score of knowledge about the CHAs’ job functions in the National Tuberculosis Control Program (NTCP); and (iii) Global Knowledge: Score of overall knowledge, which includes the arithmetic mean of the scores from components 1 and 2.*

*β = Regression coefficient; βp = Standardized regression coefficient; t = t-test; IC95% = 95% Confidence Interval; R = Reference Category; TB = Tuberculosis; DOT = Directly Observed Treatment.*

*Parameters of the models: Model 1 (global knowledge): F = 3.91; p-value = 0.002; R2 = 0.214; VIF = 1.18; Model 2 (component 1): F = 5.28; p-value < 0.001; R2 = 0.206; VIF = 1.12; Model 3 (component 2): F = 2.19; p-value = 0.0494; R2 = 0.071; VIF = 1.17*

Receiving training on tuberculosis was positively associated with global knowledge (β=6.16; p<0.001), knowledge about tuberculosis (β=9.62; p<0.001), and knowledge about the occupational functions of CHA in the PNCT (β=3.48; p=0.039) (see Table 1). Consequently, professionals who had undergone training showed 6.16%, 9.62%, and 3.48% higher global knowledge, knowledge about tuberculosis, and knowledge about their occupational functions in the PNCT, respectively, compared to CHA who had not received such training.

Lastly, access to the tuberculosis guide/manual was positively associated with global knowledge (β=3.59; p=0.026) and knowledge about tuberculosis (β=5.64; p=0.036) (refer to Table 1), indicating that CHA who reported having access to the guide demonstrated greater global knowledge and knowledge about tuberculosis.

## DISCUSSION

This study highlights that 59.1% (65 out of 110) of Community Health Agents (CHA) reported having experience in accompanying individuals with tuberculosis. This finding is similar to the results from studies conducted in other developed regions of Brazil in the context of Primary Health Care^(11,19)^. This confirms the active participation of CHA in tuberculosis control, aligning with the objectives of the National Tuberculosis Control Program (PNCT)^(4)^. However, the frequency of Directly Observed Treatment (DOT) supervisions, conducted once or twice a week by most (88.2%, 29 out of 34), does not align with the recommended frequency of five times a week, with a minimum of three times^(6)^. This discrepancy might be related to a lack of knowledge about the recommended frequency, as evidenced in previous studies^(11)^.

The activities of CHA in the PNCT^(4)^, as described in the Manual for Community Health Agents - Tuberculosis^(3)^, are expected to be based on labor practices grounded in specific knowledge, such as infection definition, clinical manifestations, classification, prevention measures, diagnosis, and treatment. However, the analysis of the evaluated questions revealed a low rate of correct responses on topics including signs and symptoms, the period of transmission of the etiological agent, the use of Personal Protective Equipment (PPE), and the treatment of pulmonary tuberculosis.

During the implementation of DOT, CHA are exposed to biological risks, especially in the first two weeks of treatment. Non-use or irregular use of protective measures can pose health risks to the professional. When inquired about precautions taken during DOT supervision, a low adherence to protective measures was observed, with 64.7% mentioning hand hygiene and only 8.8% citing the use of PFF2 (N95) respirators. These safety gaps could stem from various factors, such as limited knowledge about occupational protection, lack of access to information, unavailability of PPEs in health services, and absence of adequate guidance and supervision. Nevertheless, most of these aspects are fundamentally linked to health management, which should bear responsibility for minimizing the biological risks faced by CHA. The use of N95 masks, though emphasized during the Covid-19 pandemic^(20)^, should continue as a routine practice for CHA conducting DOT, in line with the precautions based on the transmission of *Mycobacterium tuberculosis* ^(21)^.

Regarding tuberculosis training topics, their relevance in consolidating essential knowledge for effective disease control practice is evident. However, less than half of the CHA (46.4%, 51 out of 110) reported having received specific training on tuberculosis, a figure lower than that reported in other studies^(11-12)^. This finding is significant for the municipal management of the study, as limited training for assuming the role can impact the safety and quality of actions undertaken^(22)^, leading to uncertainties about the real functions of CHA and affecting their performance^(23)^. These factors may prompt professionals to undertake actions that could be risky for themselves.

Additionally, it is important to consider that this issue might be related to the policy of training and updating CHA. The analysis of the introductory course syllabi for CHA^(24)^ shows an absence of content related to biosafety, potentially contributing to approximately 30% of CHA not mentioning tuberculosis protection measures in their training.

Similarly, in another guiding document for the training of CHAs, titled “Guidelines for the Training of Community Health Agents in Care Lines,” there is no clear reference to the topic of biosafety. Instead, it discusses a related issue: “Protection of the CHA in risk situations in their territory”^(25)^. Consequently, there is a lack of a clear definition of themes related to occupational biological safety. In our view, this should include detailed protective measures against tuberculosis and, similarly, the role of these workers in controlling the disease within the NTCP^(6)^. The absence of such details contributes to the neglect of this content. With this in mind, it is expected that the recent regulation of CHAs as health professionals will positively impact their training in terms of biological risks and preventive measures ^(26)^.

Our results confirmed that CHAs’ knowledge about tuberculosis is comprehensive and directly related to their work experience. This correlation has also been confirmed in other studies^(11-12)^. We assume that the longer CHAs work, the more opportunities they have for training and the greater likelihood they have of effectively following up with individuals with tuberculosis. Practical experience can both generate knowledge and lead to the recognition of the need to stay updated on the topic. However, practice without theoretical training, the availability of resources, and supervision can lead to the adoption of non-recommended practices, such as the infrequent use of N95 masks by CHAs during DOT monitoring.

It is important to note that the interval between one training session and the next can negatively impact the practices developed by CHAs in the communities they serve^(12)^. This underscores the importance of establishing a continuous in-service education schedule that covers tuberculosis care for the population served and occupational safety.

The inadequacy of training is evident in the CHAs’ knowledge and their performance in the NTCP, particularly when considering only their knowledge about tuberculosis (component 1), where 47.6% of CHAs demonstrated deficient knowledge. A similar finding was reported in a study conducted in Lesotho, Africa^(27)^.

The well-known consequences of limited knowledge about tuberculosis cannot be overlooked. Such limitations reinforce unsatisfactory practices in actions considered essential for successful infection control^(28-29)^, significantly interfere with the community education process for managing tuberculosis, weaken the DOT strategy, and result in low community adherence to tuberculosis control services ^(27)^. Furthermore, the knowledge and training of CHAs in tuberculosis treatment are factors that influence patient satisfaction with the services these workers provide ^(30)^.

This understanding is supported when observing that, in this study, a positive association was established between the participation of CHAs in training sessions addressing tuberculosis and their knowledge about the disease (β=9.62; p<0.001), their roles in the NTCP (β=3.48; p=0.039), as well as their overall knowledge (β=6.16; p<0.001). These findings confirm the association of satisfactory knowledge about tuberculosis with the participation of CHAs in training sessions ^(11, 31)^, a participation that has also been identified as a motivational factor in the work of CHAs^(32)^.

The aforementioned results highlight the importance of consistently conducting training sessions that address the theme of tuberculosis as a strategy for disease control. Educational processes contribute to the development of competencies in CHAs, enhancing their awareness and understanding of their responsibilities in the NTCP, as well as fostering positive changes in their work practices ^(31)^. Therefore, the in-service education process for CHAs should be a continuously implemented practice, constituting an important strategy used by family health teams to strengthen the tuberculosis control program and enhance occupational safety.

### Study Limitations

This study has some limitations. Conducted in only one health district, it does not allow for a statistical assertion that the findings apply to other districts. Additionally, the study was based on self-reports, which are subject to memory biases and social desirability. Lastly, the absence of a longitudinal component to track changes in the knowledge and practices of CHA over time did not allow for the identification of significant changes.

### Contributions to Nursing, Health, and Public Policy

This study offers valuable contributions, particularly in enhancing the performance of CHA in tuberculosis control. By highlighting the need for regular and specific training, it aims to ensure the safety of both users and workers. The findings of this study can guide the formulation of more effective public health strategies, focusing on the continuous education of CHA and promoting evidence-based practices. Practically, these findings indicate expected actions from the nursing team leader in tuberculosis control in primary health care.

## CONCLUSION

We observed a median global score of knowledge about tuberculosis in nearly half of the CHA participants in the study, which can be considered low in relation to the expected functions of these workers in tuberculosis control. Educational factors (training and access to the manual) and work experience proved to be important for the knowledge of these workers.

Considering that the participants had at least one year of experience and that less than half reported participation in training activities, it reaffirms that as the duration of service as an CHA increased, so did their global and tuberculosis-related knowledge. The low rate of hand hygiene reported during DOT supervision, the negligible use of N95 masks (98.8%), and the non-use of protective eyewear by CHA demonstrate the inadequacy of the approach to occupational safety measures against biological risk. This may reflect flaws in the policy of training and updating these workers, but also reveals shortcomings in the management of occupational biological risk.

These results highlight the need for improvements related to management in the planning and implementation of both the CHA admission process and in-service education regarding tuberculosis control, in addition to establishing service education indicators for CHA on tuberculosis, including occupational risk. While awaiting changes in these policies, it is considered that it falls to nurses, as team leaders, to include occupational biological risk in the topics of interest for tuberculosis control in the population, to provide in-service education to CHA on these topics and the indicated protective equipment, whenever possible, and to supervise their use.

Given the importance of CHA in the context of primary care, the routine expansion of their activities, and the findings of this study, there is a suggested need to expand research with this group, both in seeking strategies for the effectiveness of expected actions in different primary care programs and for the protection of these workers.

## Data Availability

https://doi.org/10.48331/scielodata.8BXPYU
